# From Biomaterials to Biological State Engineering: Reframing Advanced Wound Dressings as Adaptive Therapeutic Interfaces in Translational Medicine

**DOI:** 10.3390/cells15131230

**Published:** 2026-07-07

**Authors:** Tomasz Urbanowicz, Judyta Cielecka-Piontek, Krzysztof J. Filipiak, Anna Witkowska, Ewelina Grywalska, Mansur Rahnama, Zbigniew Krasiński

**Affiliations:** 1 Cardiac Surgery and Transplantology Department, Poznan University of Medical Sciences, ½ Dluga Street, 61-848 Poznan, Poland; 2 Department of Pharmacognosy and Biomaterials, Poznan University of Medical Sciences, Rokietnicka 3, 60-806 Poznan, Poland; 3 The Center of Postgraduate Medical Education, 99/103 Marymoncka Street, 01-813 Warsaw, Poland; 4 Department of Experimental Immunology, Faculty of Medical Sciences, Medical University of Lublin, 6 Chodźki Street, 20-093 Lublin, Poland; 5 Department of Dental Surgery, Medical University of Lublin, 6 Chodźki Street, 20-093 Lublin, Poland; 6 Department of Vascular, Endovascular Surgery, Angiology and Phlebology, Poznan University of Medical Science, ½ Dluga Street, 61-848 Poznan, Poland

**Keywords:** chronic wounds, biological memory, pathological tissue states, adaptive therapeutic interfaces, biological state engineering, systems biology, precision wound medicine, regenerative medicine, biomaterials, artificial intelligence

## Abstract

**Highlights:**

**What are the main findings?**

- Chronic wound persistence can be interpreted as the consequence of multiscale biological memory involving immune, stromal, metabolic, extracellular matrix, mechanical, and microbial networks.
- Current wound-care technologies can be organized within an evolutionary framework that progresses from passive dressings to adaptive therapeutic interfaces.

**What are the implications of the main findings?**

- Future therapies should focus on reprogramming pathological tissue states and restoring regenerative competence rather than solely supplying regenerative signals.
- Adaptive, data-driven wound-care systems may provide the foundation for precision wound medicine and next-generation regenerative therapies.

**Abstract:**

Chronic wounds remain a major global health challenge despite substantial advances in biomaterials, regenerative medicine, and wound-care technologies. Current therapeutic strategies are largely based on the assumption that chronic wounds represent impaired or incomplete healing responses and therefore require augmentation of regenerative processes. This paradigm has driven the development of increasingly sophisticated wound dressings incorporating extracellular matrix analogs, growth factors, stem cells, extracellular vesicles, biosensors, and bioelectronic components. However, the clinical impact of these innovations has often fallen short of expectations. In this review, we propose a conceptual framework intended to generate experimentally testable hypotheses rather than provide a definitive mechanistic model. Persistent alterations in immune, stromal, vascular, extracellular matrix, metabolic, mechanical, and microbial networks create interconnected feedback systems that resist transition toward regeneration. From this perspective, successful therapy requires not only stimulation of repair mechanisms but also disruption of the processes that stabilize chronicity. We discuss how advances in systems biology, immunomodulatory biomaterials, bioelectronics, artificial intelligence, and precision medicine support the emergence of adaptive therapeutic interfaces capable of sensing, interpreting, and reprogramming pathological tissue behavior. Unlike previous reviews that primarily summarize emerging wound dressings or regenerative biomaterials, this Review proposes a systems-level conceptual framework in which chronic wounds are interpreted as stable pathological tissue states maintained by multiscale biological memory. This perspective integrates biomaterials, systems biology, artificial intelligence, and tissue-state dynamics into a unified translational model that has not previously been presented in the wound-healing literature. Previous reviews have predominantly focused on the design, biological activity, or clinical performance of individual biomaterials. In contrast, the present Review proposes a systems-level framework that integrates wound biology, biological memory, tissue-state dynamics, artificial intelligence, and adaptive biomaterials into a unified conceptual model for precision wound medicine. This state-based model reframes advanced wound dressings as tools for biological state engineering and provides a translational framework for the future of chronic wound management.

## 1. Introduction: The Success of Innovation and the Persistence of Failure

Chronic wounds remain a major challenge in modern healthcare despite substantial advances in biomaterials, regenerative medicine, and wound-care technologies [1,2,3,4]. Diabetic foot ulcers, venous leg ulcers, and pressure injuries affect millions of patients worldwide and are associated with prolonged treatment, high recurrence rates, reduced quality of life, and considerable healthcare costs. Although numerous advanced wound dressings have been developed, improvements in clinical outcomes have often been less dramatic than anticipated from preclinical studies.

Over the past four decades, wound dressings have evolved from passive protective materials to increasingly sophisticated therapeutic platforms. Biomaterials are designed to mimic the extracellular matrix [5]. Growth factors are delivered to stimulate angiogenesis and cell proliferation [6]. Stem cells are introduced to enhance regenerative capacity [7,8]. Scaffolds are engineered to support tissue reconstruction [9]. Contemporary technologies incorporate bioactive molecules, extracellular matrix substitutes, stem cells, extracellular vesicles, biosensors, and bioelectronic components. These innovations have expanded the therapeutic capabilities of wound-care systems and improved our ability to interact with the wound microenvironment. Nevertheless, many chronic wounds remain resistant to treatment, highlighting a persistent gap between technological innovation and clinical effectiveness.

The chronic wound microenvironment is characterized by persistent inflammatory activation, excessive protease activity, impaired angiogenesis, fibroblast senescence, extracellular matrix degradation, biofilm persistence, altered mechanotransduction, metabolic dysregulation, and defective intercellular communication. These abnormalities interact through reciprocal feedback loops that stabilize pathological tissue behavior rather than representing isolated defects [10,11]. Consequently, even highly sophisticated biomaterials often operate in a biological environment that actively impedes regeneration.

Hydrogels, injectable gels, extracellular matrix substitutes, and cell-delivery systems successfully improve moisture balance, local drug delivery, and temporary structural support [12,13]. However, most remain fundamentally passive once implanted and generally target one or a limited number of biological pathways. Their therapeutic activity rarely adapts dynamically to evolving tissue conditions, and they have limited capacity to interrupt the multiscale feedback mechanisms that maintain chronicity [14]. These limitations provide part of the rationale for considering adaptive therapeutic interfaces capable of continuous sensing, interpretation, and state-directed intervention.

Hydrogels and injectable biomaterials are primarily designed to provide structural support, controlled drug release, or delivery of biological cargo [15]. Although these functions improve the local wound microenvironment, their biological activity generally remains predetermined after implantation and cannot autonomously adapt to evolving inflammatory, metabolic, microbial, or mechanical conditions within the wound [16,17,18]. Consequently, repeated clinical interventions are often required because therapeutic activity is not continuously adjusted to tissue-state dynamics. These limitations provide a strong rationale for developing adaptive therapeutic interfaces that integrate sensing, biological interpretation, and state-directed intervention.

A common assumption underlying much of regenerative medicine is that chronic wounds represent impaired or incomplete healing responses. Consequently, therapeutic strategies have largely focused on replacing or augmenting biological functions considered deficient, including growth-factor signaling, angiogenesis, extracellular matrix formation, and cellular regenerative capacity. While these approaches have produced important advances, they do not fully explain the remarkable persistence of chronic wounds or their tendency to recur despite apparently successful treatment.

Persistent inflammatory activation, fibroblast dysfunction, endothelial abnormalities, extracellular matrix remodeling, microbial dysbiosis, and cellular senescence can remain stable for prolonged periods and often persist despite therapeutic intervention.

**Figure 1:**
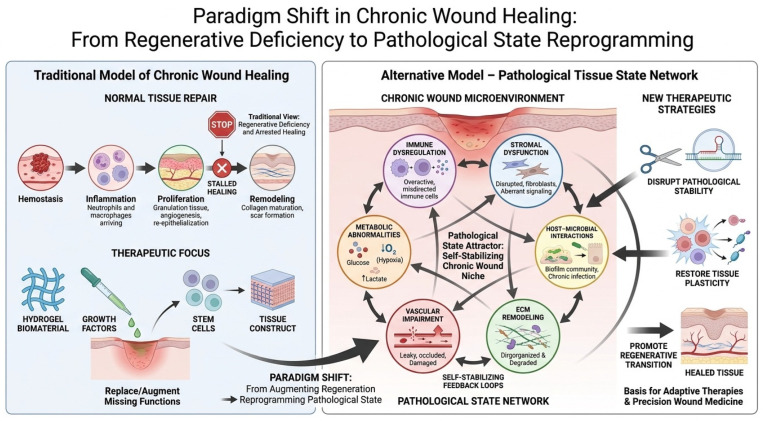
Paradigm shift in the conceptual framework of chronic wound healing. Traditional models interpret chronic wounds as regenerative deficiencies in which healing becomes arrested within the normal sequence of tissue repair. Consequently, therapeutic strategies have focused on replacing or augmenting missing biological functions through biomaterials, growth factors, cellular therapies, and tissue-engineered constructs. In the alternative framework proposed here, chronic wounds are viewed as self-stabilizing pathological tissue states maintained by interconnected networks involving immune dysregulation, stromal dysfunction, vascular impairment, extracellular matrix remodeling, metabolic abnormalities, and host–microbial interactions. These interacting processes create a stable pathological attractor that resists spontaneous transition toward regeneration. Within this model, successful therapy requires not only the enhancement of repair mechanisms but also the disruption of pathological state stability, the restoration of tissue plasticity, and the promotion of a transition toward regenerative states. The shift from regenerative augmentation to biological state reprogramming provides the conceptual basis for adaptive therapeutic interfaces and precision wound medicine. **Abbreviations:** ECM, extracellular matrix; Created by FigureLabs (Crreated by Urbanowicz, T.; 2026, ID: FL-PUB-20260602-J43PEN). **Arrow notation:** Black directional arrows indicate progression or causal transitions between biological states. Circular arrows within the pathological tissue state represent self-reinforcing feedback loops that maintain chronicity. Large arrows indicate therapeutic interventions intended to disrupt pathological stability, restore tissue plasticity, and promote transition toward regenerative healing.

In this Review, we propose that chronic wounds may be understood as stable pathological tissue states maintained by interconnected networks involving immune, stromal, vascular, metabolic, extracellular matrix, mechanical, and microbial processes. Therapeutic success depends not only on stimulating repair but also on disrupting the mechanisms that stabilize chronicity. We discuss how concepts from biological memory, systems biology, regenerative medicine, bioelectronics, and artificial intelligence can be integrated into a state-based model of wound healing and explore the implications of this perspective for the development of future wound therapies. The conceptual framework proposed in this Review is summarized in Figure 1 [19,20,21,22,23,24].

The historical evolution of wound dressings can also be interpreted in terms of their increasing capacity to interact with tissue-state dynamics (Table 1).

Finally, despite remarkable technological advances, the clinical benefit of many advanced wound dressings has remained more modest than anticipated from experimental studies. Growth factor delivery, extracellular matrix substitutes, stem-cell therapies, extracellular vesicles, and other regenerative strategies have frequently demonstrated encouraging biological activity in preclinical models, yet their effectiveness in routine clinical practice has often been inconsistent. These observations suggest that biological efficacy alone may not be sufficient to induce durable healing when pathological tissue states are maintained by multiple interacting feedback networks. Within the framework proposed in this Review, the principal limitation of many current technologies may therefore lie not in the potency of the therapeutic agent itself, but in the inability to destabilize the biological processes that maintain chronicity.

### 1.1. Literature Search Strategy and Scope of the Review

This Review was developed as a narrative, hypothesis-generating synthesis rather than a systematic review.

Publications were identified through structured searches of PubMed/MEDLINE. Although additional databases such as Scopus, Web of Science, IEEE Xplore and Embase contain complementary literature—particularly in biomaterials engineering, bioelectronics, artificial intelligence and digital health—PubMed was selected because the primary objective of this Review was to integrate biological and translational concepts rather than provide an exhaustive engineering literature survey. Consequently, relevant engineering publications not indexed in PubMed may have been underrepresented.

Predefined search terms relevant to chronic wound biology, regenerative medicine, biomaterials, immunology, systems biology, bioelectronics, artificial intelligence, and precision medicine were selected. Priority was given to original investigations, high-quality reviews, translational studies, and landmark publications that substantially advanced conceptual understanding of chronic wound pathophysiology or emerging therapeutic technologies. Studies were selected based on scientific relevance rather than predefined quantitative eligibility criteria. Additional articles were identified through citation tracking of influential publications. Because this review aims to integrate concepts across multiple disciplines rather than provide an exhaustive synthesis of evidence, formal risk-of-bias assessment and meta-analytic methodology were not performed. The principal limitation of this approach is the possibility of selection bias and unequal representation of different areas of the literature.

### 1.2. Definition of Key Concepts

Throughout this Review, four systems-biology concepts are used repeatedly. Biological memory refers to the persistence of cellular, extracellular, metabolic, microbial, or mechanical alterations after the initiating stimulus has disappeared. Pathological tissue attractor describes a stable network configuration that resists spontaneous transition toward regeneration. Tissue plasticity denotes the capacity of the wound microenvironment to reorganize into alternative biological states in response to endogenous or therapeutic signals. Biological state engineering refers to therapeutic strategies designed to modify the overall tissue-state architecture rather than individual molecular pathways.

These terms are used at different levels of evidentiary certainty. Trained immunity, metabolic memory, fibroblast persistence, extracellular matrix remodeling, biofilm stability, and mechanotransduction are experimentally supported mechanisms in chronic wound biology. In contrast, the interpretation of these processes as an integrated tissue-level pathological attractor remains a conceptual, hypothesis-generating framework. Therefore, throughout this Review, the terms ‘pathological attractor’ and ‘biological state engineering’ should be understood as systems-level interpretations intended to guide future experimental testing rather than as fully demonstrated mechanisms in human chronic wounds.

## 2. Chronicity as a Biological State Rather than a Healing Delay

The modern understanding of wound healing emerged largely from studies describing the sequential phases of tissue repair [38,39]: hemostasis is followed by inflammation, inflammation by proliferation, and proliferation by remodeling [40]. This framework has been extraordinarily influential because it provides an intuitive model through which pathological healing can be understood. Chronic wounds are commonly described as wounds that become arrested within one or more phases of this process, particularly the inflammatory phase [41]. Although this framework remains clinically valuable, it does not fully explain several characteristic features of chronic wound biology. The concept of healing arrest assumes that regenerative processes have become inadequate rather than actively redirected [42,43]. It suggests that healing has stopped because necessary regenerative processes have become inadequate or exhausted. Yet many observations from chronic wound biology point toward active maintenance rather than passive failure.

Macrophages within chronic wounds do not simply remain inflammatory because reparative signals are absent [44,45]. These cells undergo durable functional reprogramming that reinforces inflammatory phenotypes [46,47]. Fibroblasts isolated from diabetic ulcers exhibit persistent abnormalities even after prolonged culture under standardized laboratory conditions [48,49]. Endothelial cells exposed to chronic hyperglycemia retain altered behavior long after normalization of glucose levels [50,51]. Microbial communities organize into biofilm structures that actively shape host immune responses. Extracellular matrix degradation products acquire signaling properties capable of perpetuating inflammation and tissue destruction [52,53]. These phenomena suggest that chronic wounds are maintained by self-reinforcing biological networks rather than by simple regenerative insufficiency [54].

The distinction is critical because interventions designed to accelerate repair may be fundamentally different from interventions designed to destabilize pathological states. In regenerative medicine, enhancement of healing mechanisms is often sufficient. In chronic wound medicine, the primary challenge may instead involve disruption of pathological stability [55]. This perspective aligns with broader developments across systems biology. Complex biological systems frequently exhibit multiple stable states. Cellular differentiation, immune activation, cancer progression, fibrosis, and developmental patterning are increasingly understood as transitions between alternative attractor states governed by regulatory networks [56]. Once established, these states can become remarkably resistant to perturbation. Rather than viewing chronicity solely as incomplete healing, we propose an alternative interpretation: chronic wounds are tissues occupying stable pathological states.

Although diabetic foot ulcers, venous leg ulcers, and pressure injuries share many features of chronic wound biology, including persistent inflammation, extracellular matrix remodeling, impaired angiogenesis, and defective tissue repair, the biological mechanisms that initiate and sustain these processes differ considerably. Appreciating these disease-specific drivers is important because it suggests that chronicity does not arise through a single universal pathway but through distinct network configurations that converge toward a common pathological tissue state. This distinction has important implications for precision wound medicine, indicating that successful state reprogramming will likely require therapeutic strategies tailored to the dominant biological mechanisms underlying each wound phenotype in Table 2.

Although diabetic foot ulcers, venous leg ulcers and pressure injuries converge toward chronic inflammatory tissue states, they do so through distinct biological trajectories dominated by different vascular, metabolic, mechanical and microbial drivers. This heterogeneity argues against a single universal mechanism of chronicity and supports the concept that adaptive therapeutic interfaces should be tailored to disease-specific tissue-state architectures.

These disease-specific differences provide the biological context within which multiscale biological memory develops, ultimately contributing to the stabilization of chronic wound states discussed in the following section.

## 3. Biological Memory and the Persistence of Pathological States

Immune cells exhibit trained immunity, in which prior exposure to inflammatory stimuli induces persistent functional adaptations [57,58]. Fibroblasts retain epigenetic signatures of prior injury and disease states [59]. Endothelial cells demonstrate metabolic memory following hyperglycemic exposure [60]. Mechanical forces produce durable alterations in gene expression and cellular behavior. Even extracellular matrices retain structural and biochemical information that can influence future cellular responses [61,62]. In conclusion, these observations suggest that biological systems continuously accumulate information about past experiences. These observations have important implications for understanding chronic wound persistence.

A diabetic foot ulcer does not arise in a biologically naïve environment [63]. It develops within tissues shaped by years of metabolic dysfunction, vascular compromise, neuropathy, oxidative stress, and recurrent inflammatory activation [64]. Each of these processes leaves biological traces. These traces are stored through epigenetic modifications, altered cellular phenotypes, matrix remodeling, microbiome restructuring, and changes in tissue architecture [65,66]. Consequently, wound development occurs within tissues that have already undergone extensive biological adaptation. This history is not merely descriptive. It actively influences future behavior.

A central concept proposed in this Review is that chronic wounds retain pathological information across multiple levels of biological organization. We hypothesize that chronic wounds retain pathological information across multiple biological levels, including interconnected forms of biological memory involving immune cells, stromal populations, metabolic pathways, extracellular matrices, mechanical environments, and microbial ecosystems. These memory systems collectively contribute to the persistence and stability of pathological tissue states. The principal forms of biological memory relevant to chronic wound chronicity are summarized in Table 3.

Although trained immunity, metabolic memory, fibroblast persistence, extracellular matrix remodeling, mechanotransduction, and microbiome stability have each been demonstrated experimentally, direct evidence that these processes collectively generate tissue-level biological memory remains unavailable. Throughout this Review, the term biological memory therefore refers to an integrative conceptual framework rather than a single experimentally validated biological entity.

From this perspective, chronic wounds can be understood as memory-bearing biological systems whose responses to injury are constrained by accumulated pathological information [20]. Healing, therefore, requires more than the provision of regenerative stimuli. It requires modification of the biological memory that maintains chronicity.

**Figure 2:**
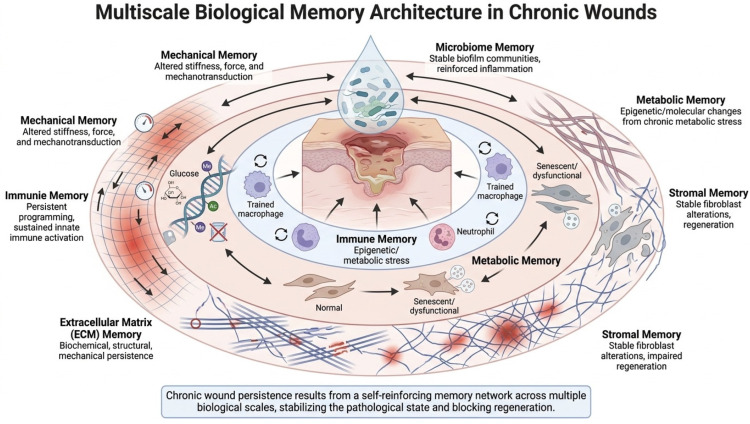
Multiscale biological memory architecture in chronic wounds. Chronic wounds can be conceptualized as biological systems that accumulate and retain pathological information across multiple interacting levels of organization. Immune memory, mediated through trained immunity and persistent inflammatory programming, promotes sustained activation of innate immune networks. Metabolic memory, particularly in diabetes, results from durable molecular and epigenetic changes induced by prolonged metabolic stress. Stromal memory arises through stable alterations in fibroblast phenotype that impair regenerative competence. Extracellular matrix memory reflects the persistence of structural, biochemical, and biomechanical information embedded within remodeled tissue architecture. Microbiome memory is maintained through stable biofilm communities and host–microbial feedback loops that reinforce chronic inflammation. Mechanical memory emerges from alterations in tissue stiffness, force transmission, and mechanotransduction pathways. Together, these interconnected memory systems generate a self-reinforcing pathological state that resists spontaneous transition toward regeneration. The persistence of chronic wounds may therefore reflect not the failure of individual repair mechanisms but the stability of a distributed memory network operating across multiple biological scales. Abbreviations: ECM, extracellular matrix. Created by FigureLabs (Created by Urbanowicz, T.; 2026, ID: FL-PUB-20260602-SRM7E9). **Arrow notation:** Solid arrows indicate the direction of biological signaling and intercompartmental communication. Opposing arrows denote reciprocal interactions between memory systems. Circular arrows represent persistent self-maintaining cellular programs. Converging arrows toward the wound illustrate the integrated contribution of multiscale biological memory to stabilization of chronic wound chronicity.

Persistent inflammation, fibroblast dysfunction, vascular impairment, extracellular matrix remodeling, microbial dysbiosis, and metabolic abnormalities are often studied as independent pathological processes [82,83]. They interact continuously across multiple biological scales, creating self-reinforcing networks that stabilize chronicity and constrain regenerative responses [84,85]. This multiscale architecture of memory is summarized in Figure 2.

## 4. Immune Memory Beyond Adaptive Immunity

For decades, immunological memory was considered the exclusive domain of adaptive immunity [86]. This paradigm has been fundamentally revised by the discovery of trained immunity [57,87]. Virtually every biological system retains information about prior experiences. Immune responses are shaped by previous encounters [88]. Fibroblasts retain signatures of earlier injuries [89,90]. Vascular cells remember metabolic insults [91].

Innate immune cells, including monocytes, macrophages, and neutrophils, are now known to undergo durable functional reprogramming following exposure to inflammatory stimuli [92,93]. Rather than returning to a neutral baseline after activation, these cells may adopt altered transcriptional and metabolic states that persist long after the original trigger has disappeared. This phenomenon has profound implications for chronic wounds. Traditional models assume that inflammatory persistence arises because wounds continuously generate inflammatory signals [94]. While this undoubtedly contributes, trained immunity introduces a complementary possibility: inflammatory cells themselves may become altered in ways that predispose them toward pathological behavior.

Macrophages isolated from diabetic wounds provide a compelling example [95]. These cells frequently exhibit persistent pro-inflammatory characteristics, including enhanced production of tumor necrosis factor-α, interleukin-1β, and reactive oxygen species. Importantly, such abnormalities often persist even when cells are removed from the wound environment and studied ex vivo. This suggests that the inflammatory phenotype has become embedded within the cells themselves. The distinction is crucial. If inflammation is driven primarily by environmental cues, then removing those cues should restore normal function. If inflammation is maintained by cellular memory, environmental modification alone may be insufficient [96]. This may partially explain why many therapies that reduce inflammatory stimuli fail to fully restore regenerative competence. The inflammatory network has already undergone biological reprogramming. In this context, chronic wounds resemble other diseases increasingly recognized as disorders of maladaptive immune memory, including atherosclerosis, chronic inflammatory bowel disease, and certain autoimmune conditions.

## 5. Metabolic Memory and the Diabetic Wound

Among all chronic wound populations, diabetic patients provide perhaps the clearest evidence for persistent biological memory. The concept of metabolic memory emerged from long-term diabetes studies demonstrating that prior periods of poor glycemic control continue to influence vascular outcomes even after glucose normalization [97]. This observation challenged the prevailing assumption that tissue damage reflects only current metabolic conditions. Subsequent investigations revealed that transient hyperglycemia induces durable epigenetic modifications affecting endothelial cells, inflammatory cells, and stromal populations [91]. These modifications alter transcriptional programs long after glucose concentrations are normalized. The diabetic wound, therefore, develops within tissues already conditioned by years of metabolic stress. Importantly, this conditioning extends far beyond vascular dysfunction. Hyperglycemia influences mitochondrial function, reactive oxygen species generation, chromatin accessibility, cytokine signaling, and cellular metabolism [98]. Collectively, these alterations create a tissue environment predisposed toward inflammation, impaired angiogenesis, and defective repair. From a translational perspective, this observation may explain why many wound-directed interventions underperform clinically. Therapies applied locally to wounds must compete against pathological programs established over years or even decades. In effect, the wound inherits the memory of systemic disease [99].

In addition to metabolic memory, diabetic wound healing is impaired by several interacting biological and clinical barriers. Peripheral neuropathy predisposes to repetitive, unnoticed trauma, while microvascular dysfunction limits oxygen and nutrient delivery [100,101]. Persistent hyperglycemia promotes the accumulation of advanced glycation end products, oxidative stress, endothelial dysfunction, impaired macrophage polarization, fibroblast senescence, defective keratinocyte migration, and reduced angiogenic signaling [102,103]. Biofilm formation and recurrent infection further reinforce inflammatory persistence, whereas impaired stem-cell mobilization limits endogenous regenerative capacity [104,105]. These interacting barriers help explain why normalization of glycemic control alone rarely restores regenerative competence.

## 6. Fibroblast Memory and the Failure of Regeneration

Few cell populations are more central to wound repair than fibroblasts [106]. These cells coordinate extracellular matrix deposition, regulate tissue architecture, influence immune responses, and contribute to angiogenic signaling. Historically, fibroblasts were viewed as relatively passive responders to environmental stimuli. Contemporary research paints a very different picture. Fibroblasts possess remarkable phenotypic plasticity. More importantly, they possess memory. Studies across fibrosis, cancer biology, and regenerative medicine have demonstrated that fibroblasts can retain stable transcriptional identities shaped by prior environmental exposures [107]. Once established, these identities may persist despite substantial changes in surrounding conditions.

In chronic wounds, fibroblasts frequently exhibit reduced proliferative capacity, impaired migration, altered extracellular matrix production, and diminished responsiveness to growth factors [82,108]. Notably, many of these abnormalities persist following isolation and culture under standardized laboratory conditions [109,110]. Such observations suggest that fibroblasts do not merely respond to pathological environments. They become pathologically programmed by them.

## 7. Extracellular Matrix as a Biological Archive

Perhaps the most underappreciated form of memory in chronic wounds resides not within cells but within the extracellular matrix [111,112]. Traditionally regarded as a structural scaffold, the extracellular matrix is increasingly recognized as a dynamic repository of biological information. Matrix architecture influences cellular behavior through mechanotransduction pathways [113]. Matrix-bound growth factors regulate signaling gradients. Matrix degradation products function as biologically active molecules capable of modulating inflammation and repair [114]. Every inflammatory episode, mechanical injury, vascular insult, and remodeling process leaves traces within matrix organization [115]. These traces influence subsequent cellular behavior, creating feedback loops that may persist for prolonged periods. Chronic wounds exhibit profound matrix abnormalities, including excessive proteolysis, altered collagen organization, accumulation of degradation products, and disruption of normal biomechanical properties [85]. These changes are often interpreted as consequences of chronicity. An equally important possibility is that they contribute to its maintenance. This perspective challenges traditional approaches to biomaterial design. Many advanced dressings attempt to replace damaged extracellular matrices with synthetic or biological analogues [116,117]. Yet if pathological matrices function as repositories of maladaptive information, therapeutic success may depend not merely on replacement but on erasure of pathological matrix memory.

## 8. Toward a Unified Theory of Chronicity

**Figure 3:**
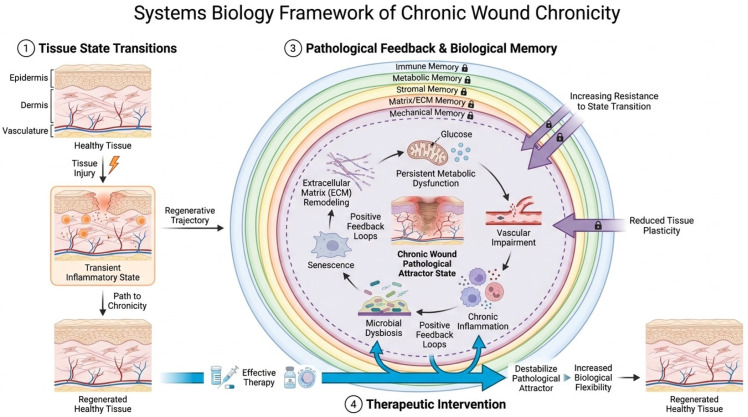
Systems biology framework of chronic wound chronicity. Biological tissues can occupy multiple stable organizational states governed by interacting cellular, molecular, metabolic, biomechanical, and ecological networks. Following injury, healthy tissues normally transition through a transient inflammatory state before returning toward a regenerative trajectory. In the presence of persistent metabolic dysfunction, vascular impairment, chronic inflammation, microbial dysbiosis, senescence, or extracellular matrix remodeling, tissues may instead enter an alternative pathological attractor characterized by stable chronicity. Within this state, multiple positive feedback loops reinforce one another, promoting persistence of inflammation, impaired angiogenesis, fibroblast dysfunction, matrix degradation, and ecological instability. Progressive accumulation of biological memory reduces tissue plasticity and increases resistance to state transition. Consequently, chronic wounds are maintained not simply by defective repair mechanisms but by the stability of an integrated pathological network. Effective therapies may therefore require destabilization of pathological attractors and restoration of biological flexibility before regenerative processes can be fully reactivated. Created by FigureLabs (created by Urbanowicz, T.; 2026, ID: FL-PUB-20260602-VBUGJQ). **Arrow notation and symbols:** Black arrows indicate the direction of biological interactions and state transitions. Circular arrows represent self-reinforcing positive feedback loops. The blue arrow denotes therapeutic reprogramming directed toward destabilization of the pathological attractor and restoration of regenerative tissue flexibility. Purple arrows indicate increasing resistance to state transition and progressive loss of tissue plasticity resulting from accumulated biological memory. Lock symbols denote the stabilization and persistence of biological memory domains, while the concentric colored rings represent the cumulative integration of multiple memory systems into a stable pathological network.

Rather than operating independently, immune, stromal, metabolic, extracellular matrix and microbial memory systems form interconnected regulatory networks. This view raises the possibility that chronic wounds may represent alternative stable states of tissue organization maintained through self-reinforcing feedback loops. The conceptual framework for this transition from distributed biological memory to pathological state stabilization is illustrated in Figure 3.

Interventions targeting single cytokines, growth factors, or cellular populations are unlikely to induce durable tissue-state transitions when pathological stability is maintained by distributed network interactions. This concept provides a mechanistic rationale for developing multimodal adaptive therapeutic interfaces that simultaneously modulate multiple biological compartments.

## 9. Pathological Attractor States: A Systems Biology Framework for Chronic Wounds

Cellular differentiation, immune adaptation, developmental patterning, fibrosis, and tumor evolution are increasingly interpreted as emergent properties arising from network organization rather than from individual molecular pathways. In each case, biological behavior is determined less by the activity of isolated components than by the structure of the interactions linking them [118,119]. Microbial dysbiosis cannot be understood in isolation from host immune activity. Instead, each process both influences and is influenced by the others through continuous reciprocal communication. Within such systems, memory becomes distributed. Pathological information is not stored within a single cellular compartment but embedded throughout an interconnected network. Inflammatory activation influences extracellular matrix composition [120]. Matrix remodeling alters mechanotransduction [121]. Mechanical signaling modifies cellular phenotype. Cellular behavior reshapes microbial ecology [122]. Microbial activity further influences inflammation. Through these interactions, biological memories become coupled across scales, generating self-reinforcing patterns of tissue behavior that persist long after the initiating insult has disappeared [123]. The persistence of chronic wounds may therefore reflect not the stability of individual pathological mechanisms but the stability of the network architecture itself.

### 9.1. Biological Attractors and Tissue-State Stability

Nonlinear feedback networks can generate stable organizational states, commonly referred to as biological attractors. Within dynamical systems theory, attractors represent preferred configurations toward which complex systems evolve and within which they tend to remain despite external perturbations. Although originally developed in mathematics and physics, attractor concepts have increasingly been applied to biological systems, including cellular differentiation, immune-cell polarization, fibrosis, and tumor evolution.

Persistent inflammation, fibroblast dysfunction, endothelial impairment, extracellular matrix remodeling, microbial dysbiosis, and metabolic abnormalities frequently coexist over prolonged periods despite substantial fluctuations in local and systemic conditions [124]. Importantly, these processes do not occur independently. Inflammatory signaling influences matrix composition and vascular function, matrix remodeling alters mechanotransduction pathways, microbial communities modulate host immunity, and metabolic dysfunction affects multiple cellular compartments simultaneously [125]. Collectively, these interactions create a densely interconnected regulatory network capable of maintaining pathological behavior over time.

Healing processes and chronicity may be interpreted not simply as different stages along a continuous repair trajectory but as alternative organizational states of the tissue microenvironment. Healthy wound repair represents a transient regenerative state that ultimately resolves toward tissue homeostasis [126]. Chronic wounds, in contrast, may occupy a distinct pathological state characterized by self-reinforcing biological interactions that limit spontaneous transition toward regeneration [127]. The concept of attractor-like tissue states, therefore, provides a theoretical basis for understanding why chronic wounds frequently persist despite interventions that successfully modify individual biological pathways.

### 9.2. Experimental Evidence Supporting Attractor-like Behavior in Chronic Wounds

One of the most prominent characteristics of attractor systems is persistence [128,129].

### 9.3. Attractor Concepts in Biological Systems

Cellular differentiation, immune-cell polarization, fibrosis, and tumor evolution have all been described within frameworks in which biological behavior is governed by network organization rather than by individual molecular pathways [130,131,132]. A defining feature of such systems is their ability to maintain stable functional identities despite ongoing environmental fluctuations [67]. In fibrosis, for example, interactions among inflammatory signaling, fibroblast activation, extracellular matrix remodeling, and mechanotransduction create self-reinforcing networks that sustain pathological behavior even after the initiating insult has diminished [133].

Although direct evidence for tissue-level attractors in chronic wounds remains limited, many characteristics of chronic wound biology—including persistent inflammatory activation, stable fibroblast dysfunction, extracellular matrix remodeling, microbial dysbiosis, and frequent recurrence—are consistent with attractor-like behavior. These observations support the hypothesis that chronicity may reflect the stability of an integrated pathological network rather than the persistence of isolated tissue repair defects.

The concept of pathological tissue states implies that chronic wound persistence is not maintained by isolated biological abnormalities but by interconnected feedback structures operating across multiple levels of organization. Immune dysregulation, extracellular matrix remodeling, vascular dysfunction, metabolic stress, cellular senescence, biomechanical alterations, and host–microbial interactions continuously influence one another through reciprocal signaling pathways. These interactions generate self-reinforcing feedback loops that stabilize pathological behavior, reduce tissue plasticity, and increase resistance to regenerative transition. Importantly, the persistence of chronic wounds may therefore arise less from the magnitude of individual pathological processes than from the architecture of the networks that connect them. From a systems perspective, these feedback structures collectively contribute to the formation and maintenance of a pathological tissue attractor. Identification of such state-stabilizing loops is particularly relevant because they represent potential points of therapeutic intervention through which chronicity may be destabilized and regenerative trajectories restored. The principal feedback mechanisms currently implicated in chronic wound persistence, together with representative biomarkers and emerging state-reprogramming strategies, are summarized in Table 4.

Importantly, the relationships between different forms of biological memory and tissue-state stabilization should not currently be interpreted as direct causal pathways. Most of the available evidence derives from reductionist experimental systems that investigate individual components of chronic wound biology. Whether these mechanisms integrate into true tissue-level attractor dynamics remains unknown and represents an important area for future investigation.

Alternative explanations for chronic wound persistence include persistent ischemia, recurrent trauma, inadequate off-loading, infection, insufficient debridement and systemic metabolic dysfunction. The tissue-state framework does not replace these mechanisms but proposes that they converge on common network configurations that can stabilize chronicity.

Moreover, the tissue-state framework should not be interpreted as a replacement for established mechanisms responsible for chronic wound persistence. In many patients, inadequate perfusion, persistent pressure, uncontrolled infection, insufficient debridement, poor glycaemic control, or suboptimal off-loading may be sufficient to explain delayed healing. Rather, we propose that these diverse clinical factors converge on common biological network configurations that subsequently acquire relative stability. Within this interpretation, the pathological tissue state represents a systems-level consequence of multiple upstream drivers rather than an independent disease mechanism. The model, therefore, complements rather than supersedes current pathophysiological concepts and provides a unifying framework through which apparently distinct causes of chronicity may be interpreted.

### 9.4. Alternative Interpretations and Boundary Conditions of the Attractor Model

The interpretation of chronic wounds as pathological tissue states stabilized by interconnected biological networks provides a coherent framework for integrating numerous observations that have traditionally been considered in isolation. Nevertheless, any systems-level model should be evaluated against the complexity and heterogeneity of clinical wound healing, where multiple biological and environmental factors interact simultaneously. The attractor framework, therefore, should not be regarded as a universal explanation for chronic wound persistence, but rather as a conceptual model that may account for a substantial subset of biological phenomena observed in chronic wounds.

An important consideration is that not all chronic wounds exhibit the degree of biological stability predicted by an attractor-like model. Many diabetic foot ulcers, venous leg ulcers, and pressure injuries heal successfully following optimization of established interventions, including effective debridement, infection control, compression therapy, pressure off-loading, vascular reconstruction, or improved metabolic management. Such observations indicate that, in at least some patients, removal of dominant pathological drivers is sufficient to restore physiological healing without requiring extensive biological reprogramming. Rather than representing a binary phenomenon, tissue-state stability is therefore more likely to exist along a continuum, with some wounds remaining relatively plastic and readily reversible, whereas others progressively acquire increasingly self-sustaining pathological characteristics that limit spontaneous recovery.

Equally importantly, delayed wound healing cannot always be attributed to intrinsic alterations within tissue-state architecture. Persistent ischemia, repetitive mechanical injury, uncontrolled infection, poor glycaemic control, malnutrition, or inadequate adherence to treatment may independently explain prolonged wound persistence in many clinical settings. Under such circumstances, chronicity may primarily reflect the continuous presence of unresolved pathogenic stimuli rather than the emergence of autonomous self-stabilizing biological networks. Distinguishing between these possibilities remains a major challenge because both mechanisms are likely to coexist and reinforce one another during disease progression.

Similar considerations apply to the concept of biological memory. Immune training, fibroblast persistence, metabolic memory, extracellular matrix remodeling, mechanical adaptation, and host–microbial interactions represent biologically distinct processes operating across different temporal and spatial scales. Each of these mechanisms has been investigated independently, and substantial experimental evidence supports their individual contribution to chronic wound biology. However, considerably less evidence demonstrates how these memory systems interact to generate an integrated tissue-level network capable of maintaining long-term pathological stability. Whether such interactions ultimately produce emergent attractor-like behavior remains an open question that requires direct experimental investigation.

Another limitation arises from the nature of the currently available evidence. Much of our understanding of chronic wound biology derives from reductionist experimental models that examine individual cellular populations or isolated molecular pathways. Macrophage polarization, fibroblast dysfunction, extracellular matrix remodeling, cellular senescence, biofilm formation, and mechanotransduction have each been extensively characterized, yet relatively few studies have simultaneously analyzed these processes within the intact tissue microenvironment over time. Consequently, the systems-level organization proposed here represents an integrative interpretation derived from multiple complementary observations rather than direct experimental demonstration of network dynamics in vivo.

From a theoretical perspective, the concept of biological attractors originates from nonlinear systems biology and mathematical modelling. Demonstrating attractor dynamics experimentally requires longitudinal, high-dimensional characterization of tissue-state trajectories rather than static molecular snapshots. Such datasets remain exceptionally limited in chronic wound research because repeated spatially resolved sampling of human wounds is technically challenging and rarely feasible in routine clinical practice. The rapid development of single-cell multi-omics, spatial transcriptomics, digital pathology, computational network modelling, and wearable biosensing technologies may eventually enable reconstruction of tissue-state transitions with sufficient temporal and biological resolution to test these concepts directly.

Rather than diminishing the value of the proposed framework, these considerations define its current boundaries and identify priorities for future investigation. The principal strength of the attractor model lies in its ability to integrate diverse pathological processes—including immune dysregulation, stromal remodeling, extracellular matrix reorganization, metabolic adaptation, vascular dysfunction, mechanobiology, and host–microbial interactions—within a common systems-level perspective (Table 5). Whether pathological tissue states represent true biological attractors or merely serve as a useful abstraction for describing highly interconnected pathological networks remains to be established. Future studies combining longitudinal clinical phenotyping with spatial multi-omics, quantitative imaging, and computational systems analysis will be essential to determine the extent to which tissue-state dynamics govern chronic wound persistence and therapeutic response.

Several clinical studies [166,167,168,169,170] demonstrate that meticulous debridement, restoration of perfusion, compression therapy, infection control, or pressure off-loading alone can successfully induce healing without requiring advanced regenerative interventions. These observations indicate that pathological tissue states likely exist along a continuum rather than representing irreversible biological attractors.

Because the attractor framework remains a hypothesis rather than a directly proven property of human chronic wounds, its value depends on whether it generates experimentally testable predictions. The following section, therefore, defines the biological, temporal, and computational criteria by which the tissue-state model could be validated or falsified.

## 10. Experimental Predictions and Validation Strategy for the Tissue-State Model

The concept that chronic wounds may represent stable pathological tissue states is ultimately valuable only if it generates experimentally testable predictions. Although direct evidence for tissue-level attractor dynamics remains limited, the proposed framework suggests several hypotheses that can be evaluated through longitudinal clinical studies, systems-biology approaches, and emerging technologies for molecular and spatial profiling.

### 10.1. Chronic Wounds Should Exhibit Reproducible Biological State Signatures

If chronic wounds occupy distinct pathological tissue states, wounds with similar underlying biology should display reproducible molecular and cellular signatures regardless of their anatomical location or clinical classification. Integrated transcriptomic, proteomic, metabolomic, and microbiome analyses should therefore identify clusters of wounds characterized by common patterns of inflammation, metabolic dysfunction, extracellular matrix remodeling, senescence, or microbial dysbiosis. Such state signatures would be expected to provide more biologically meaningful classification than conventional descriptors based solely on etiology or wound appearance.

Demonstrating tissue-state architecture will require preserving spatial biological organization rather than analyzing dissociated cells alone. Although single-cell transcriptomics has transformed our understanding of cellular heterogeneity in chronic wounds, enzymatic tissue dissociation inevitably disrupts cell–cell interactions, extracellular matrix organization, and spatial signaling gradients that may contribute to the stability of pathological tissue states. Emerging technologies, including spatial transcriptomics, multiplex imaging, imaging mass cytometry, and spatial proteomics, now permit simultaneous analysis of molecular networks within intact tissue architecture. Combined with longitudinal sampling, these approaches may provide the first opportunity to identify stable tissue-state configurations, monitor transitions between pathological and regenerative states, and experimentally validate—or refute—the systems-level framework proposed in this Review.

### 10.2. State Transitions Should Precede Clinical Healing

Within a tissue-state framework, healing is not merely the consequence of progressive repair but reflects a transition from a pathological configuration toward a regenerative one. Consequently, measurable changes in tissue-state biomarkers should occur before visible clinical improvement becomes apparent. Longitudinal studies combining molecular profiling with clinical assessment should therefore detect shifts in inflammatory activity, stromal behavior, metabolic regulation, or microbiome organization before wound closure is observed. Demonstrating such temporal relationships would provide strong support for the concept of state transition during healing.

### 10.3. Effective Therapies Should Alter Network Behavior Before Wound Closure

Traditional therapeutic evaluation often focuses on end points such as wound-size reduction or complete epithelialization. In contrast, the tissue-state model predicts that successful interventions should first modify the biological networks responsible for maintaining chronicity. Changes in inflammatory circuits, cellular senescence, extracellular matrix turnover, vascular adaptation, or host–microbial interactions should therefore precede measurable improvements in wound healing. Monitoring network-level biomarkers may provide earlier indicators of therapeutic effectiveness than conventional clinical outcomes.

### 10.4. Wound Recurrence Should Reflect Persistence of Pathological Memory

One of the most challenging features of chronic wound management is the high rate of recurrence following apparently successful healing. The tissue-state framework predicts that recurrence occurs when pathological memory systems remain incompletely resolved despite restoration of tissue integrity. Persistent molecular, cellular, matrix-associated, or microbial signatures should therefore be detectable in healed tissues that later re-ulcerate. Identification of such residual pathological states could enable development of biomarkers capable of predicting recurrence risk and guiding long-term preventive strategies.

### 10.5. Systems-Level Models Should Predict Healing More Accurately than Single Biomarkers

Because chronicity is proposed to emerge from interactions among multiple biological networks, predictive models that integrate information across biological scales should outperform approaches based on individual biomarkers. Machine-learning algorithms incorporating molecular, cellular, imaging, physiological, and clinical data should therefore demonstrate superior ability to predict healing trajectories, therapeutic responsiveness, and recurrence compared with conventional single-marker strategies. Validation of such models would support the view that tissue-state organization is a systems-level property rather than the consequence of isolated pathological abnormalities.

Collectively, these predictions provide a framework through which the tissue-state hypothesis can be evaluated experimentally. Whether chronic wounds ultimately prove to be true biological attractors or exhibit attractor-like behavior, systematic testing of these hypotheses may clarify the mechanisms responsible for chronicity and inform the development of future state-directed therapeutic strategies.

## 11. Plasticity as a Determinant of Regenerative Competence

Development, immune regulation, tissue regeneration, and environmental adaptation all require the ability of cells and tissues to modify their behavior in response to changing conditions. Plasticity [171,172] allows biological systems to transition between alternative functional states, whereas loss of plasticity constrains the range of responses that remain accessible. In this context, regeneration can be viewed not simply as the activation of repair mechanisms but as a state transition that requires sufficient biological flexibility [128,173].

The concept of plasticity therefore provides a mechanistic bridge between pathological state stability and regenerative recovery. If chronic wounds represent tissues trapped within self-reinforcing pathological configurations, then the restoration of plasticity may be a prerequisite for durable healing. Regeneration becomes not only a process of repair but also a process of reacquiring the capacity to become something different from what the tissue has previously been.

## 12. Biological State Engineering: A New Therapeutic Paradigm for Chronic Wounds

Current regenerative technologies have substantially expanded therapeutic options but generally remain focused on enhancing individual repair pathways rather than modifying tissue-state organization.

This distinction parallels conceptual developments that have occurred in several other areas of medicine. Cancer treatment has progressively evolved from targeting isolated molecular abnormalities toward reprogramming cellular states and tumor ecosystems [174]. Immunotherapy seeks not merely to eliminate pathological cells but to reshape immune-system behavior [175]. Regenerative medicine increasingly recognizes that restoration of tissue function depends on coordinated interactions among multiple biological networks rather than activation of individual pathways alone [176]. Chronic wound therapy may be approaching a similar transition.

This concept may be described as biological state engineering. Biological state engineering refers to the intentional modification of multiscale regulatory networks in order to alter the functional identity of a tissue [177,178]. In contrast to conventional regenerative approaches, which primarily supply biological inputs, state engineering seeks to influence how tissues interpret, integrate, and respond to biological information [179]. The ultimate goal is not simply enhancement of repair but restoration of adaptive tissue behavior.

Although still largely conceptual, this framework provides a unifying perspective through which diverse emerging technologies can be understood. Emerging technologies should be viewed as complementary components of adaptive therapeutic systems capable of modifying different aspects of tissue-state organization.

Several technologies already illustrate the transition toward adaptive therapeutic interfaces. Commercial wound-imaging platforms incorporating artificial intelligence can quantify wound area and tissue composition, while flexible electrochemical sensors capable of monitoring pH, oxygen tension or inflammatory biomarkers have entered early clinical evaluation. Closed-loop systems integrating sensing with autonomous drug delivery remain largely preclinical, although responsive hydrogels and electrically controlled release platforms provide proof of concept that therapeutic activity can be dynamically adjusted in response to changes in tissue state.

Beyond demonstrating biological efficacy, successful clinical implementation of adaptive therapeutic interfaces will depend on overcoming numerous practical translational barriers. Manufacturing reproducibility, long-term sterility, sensor drift, protein biofouling, calibration stability, battery life, wireless data security, reimbursement pathways, regulatory approval of combination products, patient adherence, and cost-effectiveness may ultimately determine clinical adoption as strongly as biological performance itself. Addressing these engineering, regulatory, and health-economic challenges will therefore be essential for translating adaptive wound-care platforms from promising prototypes into routine clinical practice.

## 13. Adaptive Therapeutic Interfaces: From Biomaterials to Dynamic Regulators of Tissue State

The evolution of wound dressings over the past four decades illustrates a progressive expansion in therapeutic ambition. Early dressings functioned primarily as protective barriers designed to prevent contamination and maintain a favorable local environment. Subsequent generations incorporated increasingly sophisticated biomaterials capable of regulating moisture balance, delivering bioactive molecules, supporting cellular therapies, and promoting tissue regeneration. Despite these advances, most wound technologies continue to operate as passive or semi-active platforms that deliver predefined therapeutic functions.

The concept of biological state engineering suggests a more ambitious role for future wound technologies. If chronic wounds are maintained by dynamic interactions among immune, stromal, vascular, metabolic, mechanical, and microbial networks, then therapeutic systems may need to become equally dynamic. Rather than serving solely as delivery vehicles, future wound dressings may function as adaptive therapeutic interfaces capable of continuously interacting with the evolving biological state of the tissue.

Adaptive therapeutic interfaces have the potential to enable precision wound medicine. However, their successful implementation will depend on rigorous clinical validation, regulatory approval, demonstration of cost-effectiveness, interoperability of digital platforms, and prospective trials demonstrating superiority over existing standards of care. Such systems move beyond static biomaterial design toward real-time regulation of wound behavior. Advances in biosensing technologies, flexible bioelectronics, molecular diagnostics, artificial intelligence, and responsive biomaterials increasingly make this possibility feasible.

Several emerging technologies already illustrate components of this vision. Smart dressings capable of monitoring pH, oxygen tension, temperature, inflammatory mediators, and microbial activity provide continuous information regarding wound status [180]. Responsive biomaterials can alter drug release profiles according to environmental cues. Bioelectronic devices can influence cellular behavior through electrical stimulation and modulation of endogenous signaling pathways. Machine-learning algorithms can integrate multidimensional biological data to identify patterns that may not be apparent through conventional clinical assessment [181,182]. Individually, these technologies remain developmental. Collectively, they suggest the emergence of a new class of therapeutic systems designed not merely to support healing but to actively guide tissue-state transitions.

Future wound-care platforms may increasingly combine sensing, diagnostic, and therapeutic functions within integrated systems. Their function will not be defined solely by material composition but by their capacity to continuously exchange information with the tissue environment and adjust therapeutic activity accordingly. Such systems may enable more responsive and individualized therapeutic strategies than those currently available.

Although significant scientific, engineering, regulatory, and clinical challenges remain, the convergence of biomaterials science, systems biology, bioelectronics, artificial intelligence, and precision medicine suggests that this transition is already underway. Adaptive therapeutic interfaces may therefore represent the technological embodiment of biological state engineering and provide a foundation for the next generation of regenerative therapies.

The transition from material-centered classification to function-centered classification requires a framework capable of organizing increasingly diverse therapeutic technologies according to their influence on biological behavior. Although contemporary wound-care platforms differ substantially in material composition, manufacturing methods, and technological complexity, many ultimately converge upon a limited number of biological objectives. Viewed through the lens of biological state engineering, the critical question is no longer what a technology is made of, but which component of pathological state stability it is designed to influence. This perspective enables the development of a functional taxonomy based on mechanisms of biological regulation rather than material architecture. The proposed classification system for adaptive therapeutic interfaces is summarized in Table 6 [created based on a.o.: [183,184,185]].

## 14. Precision Wound Medicine: Identifying and Targeting Tissue States

The processes that occur in chronic wounds are biologically heterogeneous. Two diabetic foot ulcers of comparable size and duration may differ substantially in their inflammatory activity, cellular senescence burden, extracellular matrix organization, vascular competence, metabolic status, or microbiome composition. Such differences may not be readily apparent through conventional clinical assessment but can profoundly influence therapeutic responsiveness.

Recent advances in systems biology offer an opportunity to address this challenge. High-throughput molecular profiling technologies now permit increasingly detailed characterization of wound microenvironments at multiple levels of biological organization. Transcriptomic analyses reveal patterns of inflammatory activation, cellular differentiation, and regenerative signaling. Proteomic approaches provide insight into extracellular matrix remodeling, protease activity, and growth-factor availability. Metabolomic profiling captures information regarding cellular energetics, oxidative stress, and metabolic adaptation. Microbiome analyses characterize ecological interactions that influence both inflammation and tissue repair. When integrated, these datasets provide a multidimensional representation of wound biology that extends far beyond traditional clinical classification systems.

Equally important are emerging spatial technologies capable of preserving tissue architecture while interrogating molecular function. Spatial transcriptomics, multiplex imaging, and single-cell approaches increasingly allow investigators to examine how cellular populations interact within their native microenvironments [186,187,188,189]. Such methods are particularly relevant to chronic wounds, where pathological behavior emerges not only from individual cell types but also from the organization of cellular and extracellular networks. The ability to map these interactions may facilitate identification of tissue states associated with healing, chronicity, or recurrence.

The integration of these complex datasets presents a substantial analytical challenge. Here, artificial intelligence and machine-learning approaches may play an increasingly important role. Chronic wounds generate large volumes of biological, clinical, imaging, and sensor-derived information that frequently exceed the capacity of traditional analytical methods. Machine-learning algorithms are uniquely suited to identifying multidimensional patterns associated with disease progression, therapeutic responsiveness, and state transitions. Rather than focusing on individual biomarkers, these approaches may enable recognition of systems-level signatures that more accurately reflect the organizational state of the tissue.

### Digital Twins and Predictive Tissue-State Modeling

An emerging extension of precision wound medicine is the concept of digital twins [190,191]. A digital twin may be understood as a computational representation of an individual wound that continuously integrates clinical, molecular, imaging, physiological, and sensor-derived information to model tissue-state dynamics in real time [192]. Rather than providing a static description of wound biology, digital twins offer the possibility of simulating disease trajectories, predicting therapeutic responses, and identifying interventions most likely to promote transition toward regenerative states [193]. As advances in biosensing, spatial omics, artificial intelligence, and adaptive therapeutic interfaces continue to converge, digital twins may evolve from predictive tools into active components of closed-loop therapeutic systems. In such a framework, treatment decisions would increasingly be guided not only by the current biological state of the wound but also by computational forecasts of its future behavior, creating new opportunities for personalized and state-directed wound care.

This perspective also has important implications for therapeutic development. Future interventions may be designed not as universally applicable products but as state-specific therapies targeted toward particular pathological configurations. Immunomodulatory strategies may be most effective in inflammation-dominated states. Senolytic approaches may benefit wounds characterized by excessive cellular senescence. Mechanobiological interventions may be appropriate for tissues exhibiting pathological matrix remodeling and stiffness. Likewise, microbiome-directed therapies may prove particularly valuable in wounds where ecological dysregulation contributes substantially to chronicity. The ability to match therapeutic mechanisms to biological state may ultimately improve both efficacy and predictability of treatment outcomes.

The convergence of systems biology, molecular diagnostics, advanced analytics, and adaptive biomaterials suggests that wound care is entering an era of precision medicine [194]. In this emerging paradigm, chronic wounds are no longer viewed as homogeneous clinical entities but as biologically distinct systems requiring individualized therapeutic strategies. Precision wound medicine therefore represents a logical extension of biological state engineering. By identifying the mechanisms that stabilize chronicity in each patient, it may become possible to design interventions that promote targeted state transitions rather than merely providing generalized support for repair [195,196]. Continued advances in molecular profiling and computational analysis are likely to influence how chronic wounds are classified and treated [197]. The transition from descriptive wound care to state-guided intervention has the potential to redefine both clinical decision-making and translational research in regenerative medicine [198].

The recognition that chronic wounds comprise biologically distinct tissue states has important implications for both diagnosis and therapeutic decision-making. While advances in molecular profiling, spatial biology, and computational analysis increasingly enable characterization of the wound microenvironment, translating this information into clinically actionable strategies remains a major challenge [199,200,201]. A state-based framework provides one potential solution by linking biological mechanisms to therapeutic objectives. Rather than selecting interventions solely on the basis of wound etiology or clinical appearance, therapies can be aligned with the dominant processes responsible for maintaining chronicity within a given tissue state [202]. Such an approach acknowledges that wounds with similar clinical characteristics may differ substantially in their underlying biology and, consequently, in their responsiveness to treatment [203]. The resulting transition from descriptive classification toward mechanism-guided intervention represents a central principle of precision wound medicine. A conceptual framework linking pathological tissue states, biological signatures, stabilizing mechanisms, and candidate therapeutic strategies is presented in Table 7.

## 15. From Biological State Recognition to Adaptive Biological Control

The preceding sections suggest that successful management of chronic wounds may require approaches that account for tissue-state dynamics. This transition represents a fundamental departure from traditional wound-care paradigms. Historically, therapeutic interventions have been designed to modify individual biological processes, such as inflammation, angiogenesis, microbial burden, or extracellular matrix remodeling. Although these approaches remain important, growing evidence suggests that chronicity emerges from interactions among multiple biological networks rather than from dysfunction within any single pathway. Consequently, effective therapies may require an equally integrated strategy.

**Figure 4:**
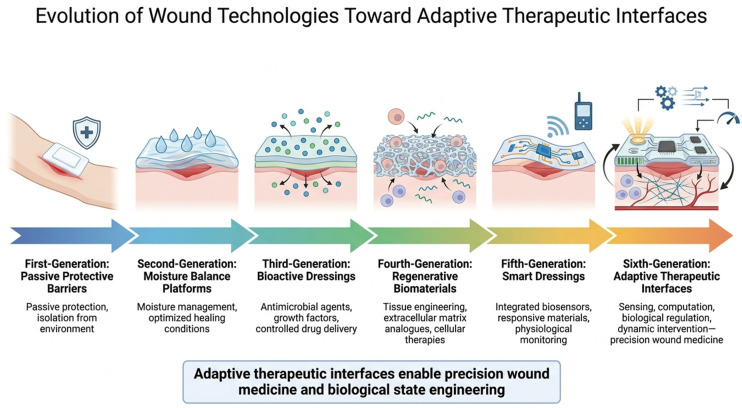
Evolution of wound technologies toward adaptive therapeutic interfaces. The historical development of wound-care technologies reflects a progressive expansion of therapeutic function. First-generation dressings served primarily as passive protective barriers that isolated wounds from the external environment. Second-generation platforms focused on moisture balance and optimization of local healing conditions. Third-generation bioactive dressings incorporated antimicrobial agents, growth factors, and controlled drug-delivery capabilities. Fourth-generation regenerative biomaterials introduced tissue-engineering concepts, extracellular matrix analogues, cellular therapies, and regenerative signaling platforms. Fifth-generation smart dressings integrated biosensors and responsive materials capable of monitoring physiological parameters and reacting to environmental changes. Emerging sixth-generation technologies function as adaptive therapeutic interfaces that combine sensing, computation, biological regulation, and dynamic intervention within integrated systems. In this framework, the therapeutic objective evolves from tissue protection toward active modulation of biological state, ultimately enabling precision wound medicine and biological state engineering. Created by FigureLabs (created by Urbanowicz, T.; 2026, ID: FL-PUB-20260602-AIUXAN). **Arrow notation:** Large gradient arrows represent the sequential evolution of wound technologies toward increasing biological sophistication and adaptability. Small black arrows indicate the direction of therapeutic delivery and biological interactions between biomaterials and the wound microenvironment. Bidirectional arrows denote reciprocal communication between the dressing and tissue. Circular arrows surrounding the adaptive therapeutic interface illustrate closed-loop sensing, computational analysis, and dynamic therapeutic feedback that continuously adjusts treatment according to tissue-state changes.

The conceptual framework proposed in Figure 4 illustrates this transition. Biological information derived from immune activity, stromal behavior, extracellular matrix architecture, vascular function, metabolic status, and microbial ecology is continuously acquired and integrated. These data collectively define the current organizational state of the tissue and provide insight into the mechanisms responsible for maintaining chronicity.

The increasing availability of molecular diagnostics, wearable biosensors, spatial profiling technologies, and advanced imaging systems creates unprecedented opportunities to characterize wound biology in real time. Importantly, the value of these technologies lies not simply in measuring individual biomarkers but in their capacity to generate multidimensional representations of tissue-state behavior. Such information may allow clinicians to distinguish wounds that appear clinically similar yet are driven by fundamentally different biological mechanisms.

Interpretation of these complex datasets will likely require computational approaches capable of identifying patterns that are not readily apparent through conventional clinical observation. Machine-learning algorithms, digital twins, and systems-level predictive models offer the potential to transform biological measurements into actionable therapeutic information. Rather than relying exclusively on static diagnostic categories, future wound management may increasingly utilize dynamic models capable of estimating tissue-state trajectories, predicting transition probabilities, and identifying mechanisms that limit regenerative progression.

Importantly, the objective of adaptive biological control is not to replace clinical judgment but to augment it. The complexity of chronic wound biology increasingly exceeds the explanatory power of isolated biomarkers and reductionist therapeutic models. By integrating information across multiple biological scales, adaptive systems may help clinicians identify hidden patterns of chronicity, anticipate therapeutic failure, and select interventions aligned with the tissue’s underlying biological state.

Viewed collectively, the progression from biomaterials to adaptive therapeutic interfaces reflects a broader transformation occurring throughout translational medicine. The focus is shifting from treating individual pathological mechanisms to regulating dynamic biological systems. In wound healing, this evolution may ultimately redefine the role of wound dressings themselves. Future dressings may no longer be regarded primarily as protective materials or drug-delivery vehicles. Instead, they may function as intelligent therapeutic interfaces capable of sensing, interpreting, and actively influencing tissue-state dynamics.

## 16. Toward a New Paradigm of Wound Healing

The history of wound-healing research has largely been defined by efforts to identify the biological mechanisms responsible for tissue repair. This approach has generated extraordinary advances in cell biology, biomaterials science, regenerative medicine, and tissue engineering. Yet despite these achievements, chronic wounds remain among the most persistent challenges in modern healthcare. The continuing gap between technological innovation and clinical outcomes suggests that our understanding of chronicity may still be incomplete.

Within such systems, pathological information becomes distributed across multiple levels of biological organization. Cellular phenotypes, extracellular matrices, metabolic programs, mechanical environments, and microbial ecosystems each retain information about previous biological events. These forms of biological memory interact continuously, generating self-reinforcing patterns of behavior that may persist long after the initiating insult has disappeared. The consequence is the emergence of a tissue state that exhibits both stability and resistance to therapeutic perturbation.

A central implication of this framework is that regeneration cannot be viewed solely as the activation of repair pathways. Regeneration also requires sufficient biological plasticity to permit state transition. Therapeutic failure may therefore arise not only from inadequate regenerative signaling but also from the progressive loss of tissue flexibility. As chronic wounds accumulate pathological memories, regenerative states may become increasingly inaccessible despite the continued presence of molecular mechanisms that support repair.

**Figure 5:**
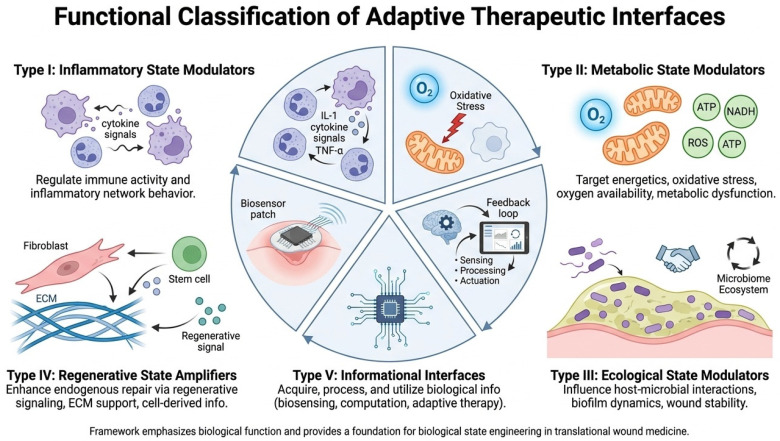
Functional classification of adaptive therapeutic interfaces. Advanced wound technologies can be organized according to their primary influence on biological state rather than their material composition. Five major functional classes emerge. Type I inflammatory state modulators regulate immune activity and inflammatory network behavior. Type II metabolic state modulators target cellular energetics, oxidative stress, oxygen availability, and metabolic dysfunction. Type III ecological state modulators influence host–microbial interactions, biofilm dynamics, and wound ecosystem stability. Type IV regenerative state amplifiers enhance endogenous repair mechanisms through regenerative signaling, extracellular matrix support, and cell-derived biological information. Type V informational interfaces acquire, process, and utilize biological information through biosensing, computational analysis, and adaptive therapeutic regulation. Although individual technologies may operate across multiple categories, this framework emphasizes biological function as the primary organizing principle and provides a conceptual foundation for biological state engineering in translational wound medicine. Abbreviations: AI, artificial intelligence; ATP, adenosine triphosphate; ECM, extracellular matrix; HIF-1α, hypoxia-inducible factor 1 alpha; IL, interleukin; NADH, nicotinamide adenine dinucleotide (reduced form); O_2_, oxygen; ROS, reactive oxygen species; TNF-α, tumor necrosis factor alpha. Created by FigureLabs (created by Urbanowicz, T.; 2026, ID: FL-PUB-20260602-HB4C9A). **Arrow notation:** Solid arrows indicate the direction of biological signaling, therapeutic activity, and intercellular communication. Bidirectional arrows represent reciprocal interactions between biological components and adaptive biomaterials. Circular arrows denote dynamic feedback loops and continuous regulation of biological processes. Blue curved arrows illustrate the integration of sensing, computation, biological interpretation, and adaptive intervention into a coordinated closed-loop therapeutic system.

Figure 5 integrates these concepts into a unified translational framework. Rather than depicting wound healing as a linear sequence of biological events, it illustrates a dynamic process in which tissue behavior emerges from interactions among memory systems, regulatory networks, and adaptive responses. Chronicity is maintained by multiscale feedback structures that stabilize pathological states, whereas successful healing requires restoration of plasticity and reorganization of network behavior. Importantly, the figure emphasizes that therapeutic interventions do not act on isolated pathways. Instead, they influence the broader architecture through which biological information is generated, stored, and interpreted.

The significance of this perspective extends beyond wound healing. Similar principles increasingly appear across diverse fields of medicine, including fibrosis, cancer biology, immunology, neurodegeneration, and metabolic disease. In each case, pathological persistence is increasingly understood as an emergent property of complex biological systems rather than the consequence of a single molecular abnormality. Chronic wounds may therefore serve as a particularly accessible model for exploring broader principles of tissue-state regulation.

The translational implications are substantial. Future therapeutic strategies may depend less on the development of increasingly potent regenerative agents and more on the ability to identify, interpret, and modify biological states. Advances in systems biology, spatial omics, biosensing technologies, artificial intelligence, bioelectronics, and adaptive biomaterials provide the foundation for this transition. Together, these approaches create the possibility of therapeutic systems capable not only of supporting tissue repair but also of guiding tissue-state transitions. Future wound-care platforms may increasingly integrate sensing, diagnostic, and therapeutic functions within a single system.

## 17. Challenges and Future Directions in Tissue-State Biology

The conceptual framework presented in this Review proposes that chronic wounds may be understood as stable pathological tissue states maintained through interconnected forms of biological memory, reduced tissue plasticity, and multiscale network interactions. While numerous observations from chronic wound biology are consistent with this interpretation, several important limitations must be acknowledged.

First, direct evidence demonstrating the existence of tissue-level attractor states in chronic wounds remains limited. Many of the concepts discussed here are supported indirectly through observations of persistent cellular phenotypes, stable inflammatory programs, extracellular matrix remodeling, microbial biofilm formation, and recurrent ulceration. Collectively, these findings suggest the presence of self-reinforcing biological organization. However, definitive demonstration of attractor dynamics will require longitudinal studies capable of capturing tissue-state transitions over time. Such investigations will likely depend upon integration of systems biology, computational modeling, and repeated molecular profiling of individual wounds throughout their healing trajectories.

Second, the biological definition of tissue states remains incompletely established. Current clinical classifications are largely based on anatomical location, etiology, and visual assessment. In contrast, a state-based framework requires identification of biological signatures that distinguish different forms of chronicity. Determining which combinations of inflammatory, stromal, vascular, metabolic, mechanical, and microbial features define specific pathological states represents a major challenge for future research. Advances in single-cell analysis, spatial transcriptomics, proteomics, metabolomics, and microbiome profiling provide powerful tools for addressing this question, but substantial work remains before such approaches can be translated into routine clinical practice.

Third, the relationship between biological memory and tissue plasticity remains incompletely understood. Although accumulating evidence supports the existence of trained immunity, metabolic memory, fibroblast memory, extracellular matrix memory, and microbiome-associated persistence, the mechanisms through which these forms of memory interact across scales are only beginning to be explored. Chronic wounds likely represent highly integrated systems in which cellular, molecular, structural, and ecological memories continuously influence one another. Understanding these interactions may prove essential for identifying therapeutic strategies capable of inducing durable state transitions.

A further challenge concerns the development of predictive models. The framework proposed here assumes that tissue behavior emerges from dynamic interactions among multiple biological networks. Yet most current experimental approaches remain reductionist in nature, focusing on individual pathways or isolated cellular populations. Future progress will require computational models capable of integrating heterogeneous biological datasets and identifying the organizational principles that govern tissue-state stability. Such efforts may ultimately enable prediction of healing trajectories, identification of transition thresholds, and recognition of patients at high risk of therapeutic failure or recurrence.

Finally, significant translational barriers remain. Precision wound medicine will require integration of advanced diagnostics, molecular profiling, biosensing technologies, artificial intelligence, and adaptive therapeutic platforms within complex clinical environments. Scientific feasibility alone will not guarantee clinical adoption. Issues related to cost, accessibility, regulatory approval, data interpretation, and implementation within routine healthcare systems will all influence the ultimate success of these approaches.

These limitations should not be viewed as weaknesses of the framework itself but rather as opportunities for future investigation. The value of a conceptual model lies not in providing definitive answers but in generating testable hypotheses. In this regard, the concepts of biological memory, tissue plasticity, state transitions, and adaptive therapeutic regulation offer a potentially productive foundation for future research. Whether chronic wounds ultimately prove to be true pathological attractors or simply exhibit attractor-like behavior, the broader challenge remains the same: understanding how complex biological systems maintain chronicity and how those systems can be redirected toward regeneration.

## 18. Conclusions

The development of advanced wound-care technologies has substantially expanded the therapeutic options available for the management of chronic wounds. Biomaterials, regenerative therapies, bioactive dressings, cellular products, biosensors, and emerging digital technologies have each contributed important advances to the field. Nevertheless, the clinical burden associated with chronic wounds remains considerable, indicating that technological progress alone is not always accompanied by proportional improvements in healing outcomes.

A recurring theme throughout chronic wound research is the persistence of common biological abnormalities, including sustained inflammation, impaired vascular function, extracellular matrix disorganization, fibroblast dysfunction, cellular senescence, metabolic disturbances, and microbial dysbiosis. Understanding how these interactions contribute to wound persistence may be as important as understanding the individual mechanisms themselves.

In this Review, we have proposed that chronic wounds can be viewed as stable biological states maintained by interacting forms of cellular, molecular, and tissue-level memory. This interpretation does not replace established concepts of wound healing, nor does it imply that chronic wounds represent true attractor states in a strict mathematical sense. Rather, it provides a systems-level framework for integrating diverse observations that are otherwise difficult to reconcile within traditional reductionist models. Within such a framework, healing may be considered not only as activation of regenerative pathways but also as the restoration of tissue plasticity and the capacity for biological reorganization.

The concepts discussed here remain largely hypothetical and require experimental validation. Future studies integrating longitudinal molecular profiling, spatially resolved analyses, computational modelling, and clinical outcome data will be needed to determine whether tissue-state dynamics can be measured, classified, and therapeutically manipulated in a clinically meaningful manner. Establishing robust biomarkers of tissue state and identifying mechanisms that govern transitions between chronic and regenerative phenotypes represent particularly important priorities.

Rather than establishing a new mechanistic model of chronic wound biology, the concept of biological state engineering should currently be regarded as an integrative systems-level hypothesis. Its principal value lies in providing a framework that connects diverse biological observations—including immune memory, stromal adaptation, extracellular matrix remodeling, metabolic programming, and microbiome dynamics—within a unified model of tissue persistence. Future experimental studies, longitudinal multi-omics analyses, spatial biology, and adaptive clinical trials will determine whether pathological tissue-state reprogramming can become a clinically actionable therapeutic strategy.

## Figures and Tables

**Table 1:** Evolution of Wound Dressings: From Passive Materials to Adaptive Therapeutic Interfaces.

Therapeutic Era	Dominant View of Chronic Wounds	Therapeutic Objective	Representative Technologies	Current Level of Evidence	Primary Biological Target	Interaction with Tissue State	Principal Limitation	Translational Significance	References
Generation I: Protective Barriers	Physical tissue damage	Isolation and protection	Gauze, cotton, bandages	Established clinical standard of care	External environment	No direct interaction	Passive function only	Established wound coverage as a therapeutic intervention	[25]
Generation II: Environmental Control	Impaired healing environment	Optimization of local conditions	Hydrogels, foams, hydrocolloids, alginates	Established clinical use supported by randomized trials and international guidelines	Moisture balance and exudate	Indirect support of repair	Limited biological specificity	Introduced microenvironment-focused wound management	[26,27,28]
Generation III: Bioactive Dressings	Deficiency of regenerative signals	Biological stimulation	Growth factors, antimicrobial dressings, ECM substitutes	Clinical use with moderate evidence; efficacy varies according to wound type and product	Inflammation and cellular signaling	Augments selected repair pathways	Variable efficacy and transient effects	Shifted dressings from passive materials to biologically active therapies	[29,30]
Generation IV: Regenerative Platforms	Loss of regenerative capacity	Restoration of tissue regeneration	Stem cells, extracellular vesicles, tissue-engineered constructs	Early clinical evidence with promising results; widespread implementation remains limited	Cellular and matrix regeneration	Enhances endogenous repair mechanisms	High complexity, cost, and manufacturing challenges	Established regenerative medicine in wound care	[31]
Generation V: Smart Therapeutic Systems	Dynamic biological dysfunction	Real-time monitoring and response	Biosensors, responsive biomaterials, wearable wound diagnostics	Early translational and pilot clinical studies; limited large-scale clinical validation	Physiological and biochemical signals	Senses tissue-state changes	Limited therapeutic autonomy	Introduced closed-loop monitoring of wound biology	[32,33]
Generation VI: Adaptive Therapeutic Interfaces	Stable pathological tissue state	Destabilization of chronicity and restoration of plasticity	AI-assisted dressings, bioelectronic platforms, adaptive biomaterials, integrated biosensor systems	Emerging translational concept supported primarily by preclinical studies and proof-of-concept technologies	Multiscale pathological networks	Continuous sensing, interpretation, and dynamic intervention	Primarily supported by preclinical studies with limited early clinical translation	Enables precision wound medicine and biological state engineering	[34,35]
Generation VII: Biological State Engineering (Proposed Future Paradigm)	Chronic wounds as self-stabilizing pathological attractors	Controlled tissue-state transition toward regeneration	Digital twins, predictive AI platforms, autonomous bioelectronic therapies, multi-modal adaptive interfaces	Hypothesis-generating future paradigm without direct clinical validation	Tissue-state architecture, biological memory, network dynamics	Active reprogramming of pathological states	Lack of experimental demonstration of tissue-state reprogramming in human chronic wounds	Provides a systems-level framework for future precision wound medicine and adaptive regenerative therapies	[36,37]

**Table 2:** Comparison of Major Chronic Wound Phenotypes Within the Tissue-State Framework based on references [38,39,40,41,42,43,44,45,46,47,48,49,50].

Feature	Diabetic Foot Ulcer (DFU)	Venous Leg Ulcer (VLU)	Pressure Injury (PI)
Primary initiating driver	Chronic hyperglycaemia, neuropathy, peripheral arterial disease	Chronic venous hypertension and venous reflux	Sustained pressure, shear forces and ischemia–reperfusion injury
Dominant vascular abnormality	Microvascular dysfunction and impaired perfusion	Venous congestion, leukocyte trapping and oedema	Local ischemia followed by reperfusion injury
Major metabolic component	Pronounced metabolic dysregulation and oxidative stress	Limited systemic metabolic contribution	Secondary metabolic disturbances related to tissue ischemia
Predominant inflammatory characteristics	Persistent innate immune activation, impaired macrophage transition, trained immunity	Chronic leukocyte recruitment, endothelial activation and sustained inflammatory signalling	Sterile inflammation induced by repetitive mechanical tissue damage
Fibroblast and stromal alterations	Senescence, impaired migration, reduced responsiveness to growth factors	Persistent fibroblast activation with excessive matrix remodelling	Impaired matrix repair secondary to repetitive tissue injury
Extracellular matrix abnormalities	Excessive proteolysis, advanced glycation end-product accumulation, abnormal collagen turnover	Matrix degradation associated with chronic inflammation and venous hypertension	Mechanical disruption with impaired matrix regeneration
Mechanical contribution	Altered plantar biomechanics due to neuropathy	Usually secondary	Primary pathogenic mechanism
Microbiological characteristics	Frequent polymicrobial biofilms and recurrent infection	Biofilms common but often less dominant	Biofilm formation frequently accompanies advanced tissue necrosis
Predominant form of biological memory	Metabolic and immune memory	Vascular and inflammatory memory	Mechanical and extracellular matrix memory
Likely priority therapeutic target	Metabolic reprogramming, immune modulation, angiogenesis	Restoration of venous function and inflammatory resolution	Pressure relief, mechanobiological modulation and tissue off-loading
Implications for adaptive therapeutic interfaces	Continuous metabolic and inflammatory monitoring	Monitoring of tissue perfusion, oedema and inflammatory activity	Real-time assessment of pressure distribution, tissue deformation and early ischemia

**Table 3:** Multiscale Biological Memory Mechanisms in Chronic Wounds.

Memory Type	Biological Substrate	Evidence in Chronic Wounds	Consequence for Healing	Therapeutic Opportunities	References
Immune memory	Trained immunity, macrophage reprogramming	Persistent inflammation	Failure of resolution	Immunomodulation	[67,68]
Metabolic memory	Epigenetic/metabolic changes	Diabetic wounds	Impaired angiogenesis	Metabolic reprogramming	[69,70]
Stromal memory	Fibroblast phenotype stabilization	Reduced regenerative competence	ECM dysfunction	Fibroblast reprogramming	[71,72,73]
ECM memory	Matrix architecture and signaling	Persistent pathological signaling	Tissue rigidity	Matrix remodeling	[74,75,76]
Mechanical memory	Mechanotransduction pathways	Abnormal stiffness	Impaired adaptation	Mechanobiology-based therapies	[77,78,79]
Microbiome memory	Biofilms and host–microbe interactions	Chronic inflammation	State stabilization	Ecological modulation	[80,81]

Abbreviations: ECM—extracellular matrix.

**Table 4:** Pathological State–Stabilizing Feedback Loops in Chronic Wounds.

Feedback Loop	Major Biological Components	Mechanism of State Stabilization	Representative Biomarkers/Readouts	Consequence for Healing	Potential State-Reprogramming Strategies	References
Inflammation–ECM Degradation Loop	Macrophages, neutrophils, MMPs, ECM fragments	Chronic inflammation increases protease activity; ECM degradation releases DAMPs that further activate inflammation	TNF-α, IL-1β, IL-6, MMP-9, TIMP-1, ECM fragments	Persistent inflammatory state and tissue destruction	Immunomodulatory biomaterials, macrophage reprogramming, MMP regulation	[134,135]
Hypoxia–Vascular Dysfunction Loop	Endothelial cells, microvasculature, hypoxia signaling	Reduced perfusion causes hypoxia; hypoxia impairs angiogenesis and further worsens vascular function	HIF-1α, VEGF, tissue oxygen tension, microvascular density	Impaired angiogenesis and nutrient delivery	Oxygen-generating biomaterials, angiogenic therapies, vascular bioelectronics	[136,137,138]
Metabolic Stress–Inflammation Loop	Hyperglycemia, mitochondria, immune cells	Metabolic dysfunction increases oxidative stress and inflammatory activation; inflammation worsens metabolic impairment	HbA1c, ROS, AGEs, NF-κB activation, mitochondrial markers	Reduced regenerative competence	Metabolic reprogramming, mitochondrial therapies, antioxidant systems	[139,140,141]
Senescence–Inflammation Loop	Senescent fibroblasts, immune cells	SASP factors perpetuate inflammation and induce neighboring-cell dysfunction	p16INK4a, p21, SA-β-gal, IL-6, IL-8	Reduced tissue plasticity and regenerative capacity	Senolytics, senomorphics, immune-mediated senescent-cell clearance	[142,143]
Fibroblast–ECM Memory Loop	Fibroblasts, collagen matrix, mechanotransduction pathways	Pathological ECM promotes abnormal fibroblast behavior; fibroblasts further reinforce pathological matrix deposition	Collagen I/III ratio, fibronectin, YAP/TAZ, α-SMA	Persistent stromal dysfunction	ECM remodeling, mechanobiological therapies, fibroblast reprogramming	[144,145,146]
Biofilm–Inflammation Loop	Microbial biofilms, innate immune system	Biofilms sustain inflammatory signaling; inflammation promotes ecological conditions favoring biofilms	Biofilm burden, bacterial diversity, quorum-sensing molecules	Chronic infection-like state and delayed healing	Anti-biofilm therapies, microbiome modulation, ecological engineering	[147,148]
Mechanical Stress–Inflammation Loop	ECM stiffness, fibroblasts, immune cells	Increased tissue stiffness alters mechanotransduction, promoting inflammation and fibrosis	Tissue stiffness, FAK activation, YAP/TAZ signaling	Reduced adaptability and tissue flexibility	Mechanomodulatory biomaterials, off-loading, force-responsive therapies	[40,149]
Microbiome–Metabolism Loop	Microbial communities, metabolites, immune cells	Microbial dysbiosis alters metabolic signaling; altered metabolism reshapes microbial ecology	Short-chain fatty acids, microbial metabolites, microbiome diversity indices	Ecological instability and chronicity	Microbiome engineering, metabolite-directed therapies	[150,151,152]
Integrated Pathological Attractor Network	Immune, stromal, vascular, metabolic, ECM, microbial systems	Multiple feedback loops interact and reinforce one another, creating a stable pathological state	Multi-omics state signatures, AI-derived state scores	Resistance to therapeutic perturbation and recurrence	Adaptive therapeutic interfaces, AI-guided precision interventions, biological state engineering	[153]

Abbreviations: AGEs, advanced glycation end products; AI, artificial intelligence; DAMPs, damage-associated molecular patterns; ECM, extracellular matrix; FAK, focal adhesion kinase; HbA1c, glycated hemoglobin; HIF-1α, hypoxia-inducible factor 1 alpha; IL-1β, interleukin-1 beta; IL-6, interleukin-6; MMP-9, matrix metalloproteinase-9; NF-κB, nuclear factor kappa B; ROS, reactive oxygen species; SA-β-gal, senescence-associated β-galactosidase; SASP, senescence-associated secretory phenotype; TIMP-1, tissue inhibitor of metalloproteinases-1; TNF-α, tumor necrosis factor alpha; VEGF, vascular endothelial growth factor; YAP/TAZ, Yes-associated protein/transcriptional coactivator with PDZ-binding motif; α-SMA, alpha-smooth muscle actin.

**Table 5:** Current Evidence Supporting the Biological State Framework of Chronic Wounds and Key Knowledge Gaps.

Biological Concept	Current Experimental Evidence	Major Limitations	Technologies Required for Validation	Translational Implications	References
Trained immunity	Persistent macrophage and monocyte reprogramming demonstrated in diabetic wounds, chronic inflammation, and metabolic disease	Most studies examine isolated immune cells rather than intact tissue ecosystems	Single-cell transcriptomics, immune lineage tracing, spatial proteomics	Identification of patients likely to benefit from immunomodulatory biomaterials	[154]
Metabolic memory	Long-lasting endothelial dysfunction and epigenetic alterations following hyperglycaemia are well established	Limited direct evidence linking metabolic memory with wound-state transitions	Epigenomics, metabolomics, longitudinal tissue sampling	Personalized metabolic interventions before advanced wound therapy	[155]
Fibroblast memory	Stable fibroblast phenotypes persist ex vivo and contribute to fibrosis and impaired regeneration	Mechanisms maintaining long-term fibroblast identity remain incompletely understood	Single-cell multi-omics, chromatin accessibility profiling	Fibroblast-directed regenerative therapies	[156,157]
Extracellular matrix memory	Matrix composition regulates immune-cell recruitment, mechanotransduction, and stem-cell behaviour	Difficult to distinguish cause from consequence of chronicity	Spatial proteomics, matrix imaging, biomechanical mapping	Rational design of next-generation biomaterials	[75,158]
Mechanical memory	Persistent changes in YAP/TAZ signalling and mechanotransduction influence tissue repair	Few longitudinal studies in human chronic wounds	High-resolution elastography, computational biomechanics	Mechanically adaptive wound dressings	[159,160]
Microbiome persistence	Stable biofilm communities contribute to inflammatory maintenance and delayed healing	Functional host–microbiome interactions remain poorly characterized	Spatial metagenomics, metabolomics, AI-based ecological modelling	Precision microbiome modulation	[161,162]
Integrated tissue-state networks	Multiple studies demonstrate interactions between immune, stromal, vascular and microbial compartments	Direct evidence for emergent tissue-state dynamics remains lacking	Spatial multi-omics, digital pathology, network biology	Biological state engineering and adaptive therapeutic interfaces	[163,164]
Pathological tissue attractors (proposed concept)	Supported indirectly by systems biology principles and convergence of multiple pathological pathways	No direct demonstration of attractor dynamics in human wounds	Longitudinal spatial multi-omics, digital twins, AI trajectory modelling	Precision wound medicine based on tissue-state transitions	[163,165]

**Table 6:** Functional Taxonomy of Adaptive Therapeutic Interfaces for Biological State Engineering *.

Primary Biological State Target	Principal Mechanism of Action	Representative Technologies	Key Biomarkers/Inputs	Therapeutic Outputs	Expected Effect on Tissue State	Translational Readiness	Primary Biological State Target
Type I: Inflammatory State Modulators	Persistent inflammatory state	Reprogramming immune-cell behavior and promoting resolution	Immunomodulatory biomaterials, macrophage-polarizing materials, cytokine-responsive dressings	TNF-α, IL-1β, IL-6, M1/M2 macrophage ratio, protease activity	Controlled release of anti-inflammatory agents, immune reprogramming signals	Reduction in inflammatory-state stability and restoration of resolution pathways	Early clinical–clinical
Type II: Metabolic State Modulators	Metabolic dysfunction and hypoxia	Restoration of bioenergetic homeostasis and oxygen balance	Oxygen-generating dressings, metabolic regulators, ROS-responsive materials, mitochondrial therapies	Oxygen tension, ROS levels, lactate, glucose, HIF-1α activity	Oxygen delivery, metabolic modulation, antioxidant responses	Increased cellular adaptability and regenerative competence	Preclinical–early clinical
Type III: Ecological State Modulators	Microbial dysbiosis and biofilm-driven chronicity	Restructuring wound ecology and host–microbe interactions	Anti-biofilm systems, microbiome-directed therapies, bacteriophage platforms, quorum-sensing inhibitors	Biofilm burden, microbial diversity, bacterial metabolites	Ecological regulation, targeted antimicrobial action	Destabilization of chronic inflammatory–microbial feedback loops	Preclinical–clinical
Type IV: Regenerative State Amplifiers	Impaired regenerative signaling	Enhancement of endogenous repair programs	Stem cells, extracellular vesicles, growth-factor platforms, ECM substitutes	Angiogenic markers, proliferative activity, matrix-remodeling indices	Delivery of regenerative signals and biological information	Amplification of regenerative trajectories	Clinical
Type V: Informational Interfaces	Incomplete biological-state recognition	Acquisition and processing of wound-state information	Biosensors, wearable diagnostics, spatial profiling systems, AI-assisted analytics	Multi-omics data, physiological parameters, imaging signatures	Real-time state assessment and prediction	Improved precision in therapeutic decision-making	Early clinical
Type VI: Adaptive Control Interfaces	Stable pathological tissue attractors	Closed-loop regulation of tissue-state dynamics	Smart dressings integrating sensors, computation, bioelectronics, and responsive drug delivery	Continuous multidimensional state monitoring	Dynamic and autonomous therapeutic adjustment	Promotion of state transition toward regeneration	Emerging
Type VII: State-Engineering Platforms (Future Concept)	Integrated pathological-state architecture	Coordinated modification of multiscale biological networks	AI-guided therapeutic systems, digital twins, adaptive bioelectronic-biomaterial hybrids	Integrated systems-level state signatures	Personalized state-transition interventions	Active reprogramming of tissue identity and restoration of plasticity	Conceptual

* This classification is proposed by the authors and synthesized from literature on wound-healing biology, regenerative medicine, systems biology, bioelectronics, biosensing technologies, and precision medicine, rather than adapted from a previously published taxonomy.

**Table 7:** Precision Wound Medicine Framework: Matching Pathological Tissue States to State-Targeted Therapeutic Strategies.

Dominant Tissue State	Defining Biological Features	Representative Biomarkers	Major Stabilizing Mechanisms	Preferred Therapeutic Interface Class *	Candidate State-Reprogramming Strategies	Predicted Clinical Goal	References
Inflammatory-Dominant State	Persistent innate immune activation, protease excess, impaired resolution	TNF-α, IL-1β, IL-6, MMP-9, neutrophil burden	Immune memory, inflammation–ECM degradation loop	Type I: Inflammatory State Modulators	Macrophage reprogramming, immunomodulatory biomaterials, protease control	Resolution of chronic inflammation	[204,205]
Metabolic-Hypoxic State	Hyperglycemia-associated dysfunction, oxidative stress, impaired perfusion	HbA1c, ROS, HIF-1α, tissue oxygen tension, VEGF	Metabolic memory, hypoxia–vascular dysfunction loop	Type II: Metabolic State Modulators	Oxygen-generating materials, metabolic reprogramming, mitochondrial therapies	Restoration of tissue bioenergetics and angiogenesis	[206,207]
Senescence-Dominant State	Accumulation of dysfunctional cells with SASP activity	p16INK4a, p21, SA-β-gal, IL-6, IL-8	Senescence–inflammation loop	Type I/II	Senolytics, senomorphics, immune-mediated senescent-cell clearance	Recovery of tissue plasticity	[208,209]
Stromal-Matrix Dysfunction State	Fibroblast dysfunction, pathological ECM remodeling, excessive stiffness	Collagen I/III ratio, fibronectin, α-SMA, YAP/TAZ	Fibroblast–ECM memory loop, mechanical feedback	Type IV: Regenerative State Amplifiers	ECM remodeling, mechanobiological therapies, fibroblast reprogramming	Restoration of regenerative competence	[210,211]
Ecological-Dysbiosis State	Biofilm persistence, microbial instability, chronic host–microbe activation	Biofilm burden, microbial diversity, quorum-sensing markers	Biofilm–inflammation loop	Type III: Ecological State Modulators	Anti-biofilm systems, bacteriophages, microbiome engineering	Ecological stabilization and inflammation reduction	[212,213,214]
Mechanobiological Dysfunction State	Pathological stiffness, altered force transmission, impaired adaptation	Tissue stiffness, FAK activation, YAP/TAZ signaling	Mechanical stress–inflammation loop	Type II/IV	Off-loading, mechanomodulatory biomaterials, force-responsive systems	Restoration of adaptive tissue mechanics	[215,216]
Mixed Multifactorial State	Multiple overlapping pathological programs	Composite molecular signatures	Integrated pathological attractor network	Type VI: Adaptive Control Interfaces	Combination therapies guided by biological-state monitoring	Destabilization of chronicity	[173]
Personalized State-Transition Target	Patient-specific systems-level state profile	Multi-omics signatures, AI-derived state scores	Network-level pathological stability	Type VII: State-Engineering Platforms	AI-guided adaptive intervention, digital twins, closed-loop therapeutic regulation	Directed transition toward regenerative state	[217,218,219]

* Interface classes correspond to the functional taxonomy presented in Table 4. Abbreviations: ECM, extracellular matrix; HIF-1α, hypoxia-inducible factor 1 alpha; IL-1β, interleukin-1 beta; IL-6, interleukin-6; ROS, reactive oxygen species; TNF-α, tumor necrosis factor alpha.

## Data Availability

No new data were created or analyzed in this study.

## Abbreviations

The following abbreviations are used in this manuscript:

AGEs	Advanced Glycation End Products
AI	Artificial Intelligence
ECM	Extracellular Matrix
FAK	Focal Adhesion Kinase
HbA1c	Glycated Hemoglobin
HIF-1α	Hypoxia-Inducible Factor 1 Alpha
IL-1β	Interleukin-1 Beta
IL-6	Interleukin-6
IL-8	Interleukin-8
M1/M2	Classically Activated/Alternatively Activated Macrophage Phenotypes
MMP	Matrix Metalloproteinase
MMP-9	Matrix Metalloproteinase-9
NF-κB	Nuclear Factor Kappa B
ROS	Reactive Oxygen Species
SA-β-gal	Senescence-Associated β-Galactosidase
SASP	Senescence-Associated Secretory Phenotype
TIMP-1	Tissue Inhibitor of Metalloproteinases-1
TNF-α	Tumor Necrosis Factor Alpha
VEGF	Vascular Endothelial Growth Factor
YAP	Yes-Associated Protein
TAZ	Transcriptional Co-Activator with PDZ-Binding Motif
α-SMA	Alpha-Smooth Muscle Actin
